# The Yin and Yang of Nrf2-Regulated Selenoproteins in Carcinogenesis

**DOI:** 10.1155/2012/486147

**Published:** 2012-05-08

**Authors:** Regina Brigelius-Flohé, Mike Müller, Doris Lippmann, Anna Patricia Kipp

**Affiliations:** Department Biochemistry of Micronutrients, German Institute of Human Nutrition, Potsdam-Rehbruecke, Arthur-Scheunert-Allee 114-116, 14558 Nuthetal, Germany

## Abstract

The NF-E2-related factor-2 (Nrf2) is a transcription factor which regulates the major cellular defense systems and thereby contributes to the prevention of many diseases including cancer. Selenium deficiency is associated with a higher cancer risk making also this essential trace element a promising candidate for cancer prevention. Two selenoproteins, thioredoxin reductase-1 (TrxR1) and glutathione peroxidase-2 (GPx2), are targets for Nrf2. Selenium deficiency activates Nrf2 as does a TrxR1 knockout making a synergism between both systems plausible. Although this might hold true for healthy cells, the interplay may turn into the opposite in cancer cells. The induction of the detoxifying and antioxidant enzymes by Nrf2 will make cancer cells chemoresistant and will protect them against oxidative damage. The essential role of TrxR1 in maintaining proliferation makes its upregulation in cancer cells detrimental. The anti-inflammatory potential of GPx2 will help to inhibit cancer initiation and inflammation-triggered promotion, but its growth supporting potential will also support tumor growth. This paper considers beneficial and adverse consequences of the activation of Nrf2 and the selenoproteins which appear to depend on the cancer stage.

## 1. Introduction

An adequate-to-high selenium supply and activation of Nrf2 by dietary compounds are considered to substantially help to prevent cancer development. Selenium exerts its effects mainly as part of selenoproteins with redox functions, and Nrf2 upregulates enzymes of the adaptive response. Thus, both systems are involved in the equipment of cells with a network of enzymes which are supposed to counteract the transformation of healthy into cancer cells by oxidative damage. However, not all attempts to prevent cancer by respective dietary supplementation/intervention ended up with a beneficial outcome; even harmful effects were observed.

The so-called Linxian trial was among the first large randomized, double-blind, primary prevention studies investigating a putative prevention of cancer by vitamins and trace elements. A mixture of selenium, vitamin E, and *β*-carotene, called factor D, significantly reduced total mortality, total cancer mortality, and most significantly mortality from gastric cancer [1]. Although selenium was not given as a single component, according to subsequent studies it appeared to have the most efficient effects [2–4]. 10 years after completion of the Linxian trial, reduction in mortality remained 5% for total and 11% for gastric cancer [5]. Considering age, the effect of factor D was much stronger in individuals younger than 55 but almost absent in subjects older than 55 years. The effect on esophageal cancer was even reversed by age [5]. The findings may indicate that selenium supplementation is only helpful to rescue a marginal deficiency and that a benefit of the supplementation depends on the stage of carcinogenesis. Whereas selenium appears to prevent initiation of cancer in healthy cells at young age, in the elderly it may be harmful and rather support tumor growth of already initiated cells [5].

**Figure 1:**
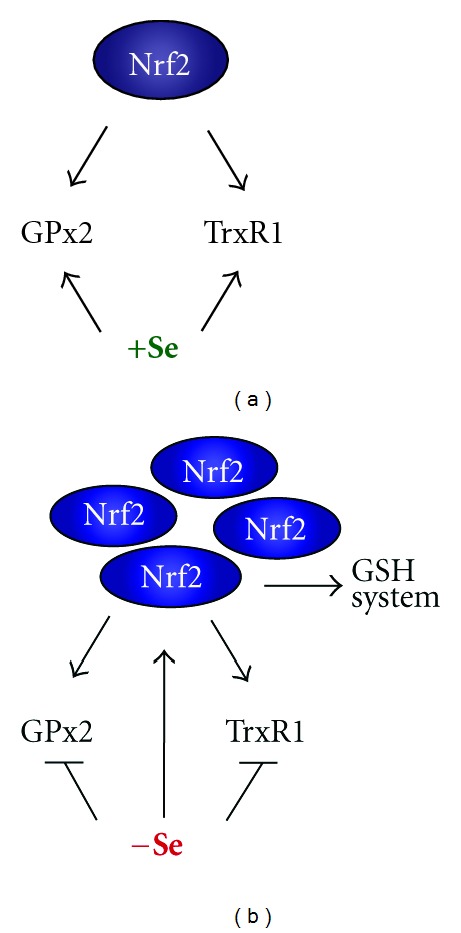
Interplay between Nrf2 and the selenoproteins thioredoxin reductase-1 (TrxR1) and glutathione peroxidase-2 (GPx2). (a) In the presence of selenium, the activation of Nrf2 leads to increased mRNA of both enzymes which can be translated into respective proteins. (b) Under selenium deficiency, Nrf2 is activated which in principle can lead to an induction of TrxR1 and GPx2 mRNA. Due to lack of selenium, the proteins cannot be synthesized. Decrease in TrxR1 further activates Nrf2, which subsequently upregulates enzymes of the glutathione (GSH) system. These, in part at least, can compensate the reduced TrxR1 activity. For details see text.

Nrf2 as regulator of the endogenous response system has generally been considered as beneficial, too. Since two selenoproteins, thioredoxin reductase-1 (TrxR1) and glutathione peroxidase-2 (GPx2), are induced by Nrf2, a synergism of both systems has been proposed [6]. Whereas this might hold true for healthy cells, solely beneficial functions of Nrf2 have been questioned especially in cancer. Recent data revealed a “dark” side of Nrf2. Its upregulation in cancer cells provides an advantage for these cells to grow and, in addition, makes them resistant against chemotherapy (reviewed in [7, 8]). Thus, also a benefit of Nrf2 activation might depend on the cancer stage. Evidence to support this idea is summarized in view of the mutual regulation of selenium/selenoproteins and Nrf2 (see Figure 1). 

## 2. Nrf2

Nrf2 is a transcription factor which is kept in the cytosol by Keap1. Keap1 acts as substrate adaptor for the Cul3-Rbx1 E3 ligase which ubiquitylates Nrf2 for proteasomal degradation. Dissociation of this complex is achieved by thiol modification of Keap1 preventing degradation and allowing newly synthesized Nrf2 to translocate into the nucleus. There it binds to the antioxidant/electrophile responsive element (ARE/EpRE) in the promoter region of its target genes. The mechanism of activation is complex but has become relatively clear in the very recent years and is described in multiple reviews [9–18].

Nrf2 regulates the expression of proteins that collectively favour cell survival. These comprise enzymes that directly or indirectly have antioxidant functions, are molecular chaperones and proteins that enhance glutathione synthesis and regeneration, belong to enzymes of the phase 2 detoxification drug metabolism systems, and recognize, repair, and remove damaged proteins and DNA. Also proteins that regulate the expression of other transcription factors, growth factors, and receptors and inhibit cytokine-mediated inflammation and autophagy are targets of Nrf2 (reviewed in [9, 19]). The realm of Nrf2 activators comprises endogenous signaling molecules produced during normal oxygen metabolism and under inflammation or other stress situations. Among activators are H_2_O_2_, ROOH, ONOO^−^, oxoaldehydes, and ketones, or cyclopentenones, like 15-deoxy-Δ^12,14^-prostaglandin J_2_. Exogenous activators comprise dietary isothiocyanates, thiocarbamates, trivalent arsenicals, quinones, dithiolethiones, vicinal dimercaptanes, certain statins, and heavy metals [9, 12, 19]. Activation by the later group allows the conclusion that these compounds may act via initiation of a moderate oxidative/electrophilic stress.

The protective role of Nrf2 is also demonstrated by genetic ablation. Nrf2-deficient mice are more susceptible to carcinogen-induced cancers [20] and develop more severe intestinal inflammation and a higher number of aberrant crypts than controls upon dextran sulfate sodium treatment [21–23]. These findings indicate a role of Nrf2 in the prevention of carcinogenesis, especially if inflammation triggered. In contrast, without challenge, Nrf2-deficient mice did not show an obvious phenotype [24]. The Nrf2 system, thus, appears to be an emergency device that comes into play if a stress is severe enough that it can no longer be handled by constitutively active systems. An enhanced Nrf2 activity by moderate stress makes cells resistant to a subsequent more severe oxidative and electrophilic stress and, thus, works like a vaccination. This way, by upregulation of defense systems, Nrf2 can prevent cancer initiation by elimination of reactive oxygen species and detoxification of carcinogens.

However, also this coin has two sites, since Nrf2 activation may not be beneficial under all circumstances. Not only normal but also tumor cells may benefit from the protective function of Nrf2 as evidenced by an increase of Nrf2 and its targets in many cancer cell lines (reviewed in [7, 17]). The physiological result of the upregulation, that is, inhibition of apoptosis and autophagy, and increase of proteasomal degradation of damaged proteins, provides a superior survival chance also for tumors. Accordingly, Nrf2 was expressed in a significantly higher proportion of endometrial serous carcinoma, the most aggressive subtype of endometrial cancer [25]. Nrf2 knockdown inhibited tumor growth from human cervical carcinoma cells in xenograft studies [26] and increased efficiency of chemotherapy in mice bearing subcutaneous tumors of these cells. In an urethane-induced lung cancer mouse model, Nrf2 deletion decreased tumorigenesis and facilitated death of early initiated cells by apoptosis [27]. These findings support cell survival properties of Nrf2 also in cancer cells.

A constitutive activity of Nrf2 is often reached by mutations in Keap1 and Nrf2 genes themselves [28]. Somatic mutations and loss of heterozygosity of Keap1 were first found in small cell lung carcinoma cell lines [29] and in non-small-cell lung cancers [30] associated with an upregulation of Nrf2 and respective target genes. RNAi-mediated downregulation of Nrf2 in these cells suppressed tumor growth in xenograft experiments and increased sensitivity to chemotherapy [31]. Somatic Keap1 mutations have also been found in cancers of gallbladder and hepatic bile duct [32] and prostate [33]. Nrf2 missense mutations were identified in lung cancers [34] and in squamous cell carcinomas of oesophagus and skin [35]. The mutations lie in the Nrf2-Keap1 interaction area, which might similarly disturb the Nrf2/Keap1 complex formation as do mutations in the same areas of Keap1 [28].

A Keap1-independent increase in the basal Nrf2 level by oncogenic alleles of *Kras, Braf*, and *cMyc* has been described recently [36]. Whereas an ectopic transduction of mouse embryonic fibroblasts (MEFs) and NIH3T3 fibroblasts with K-Ras^G12D^ led to an increase in the production of reactive oxygen species (ROS), inducible expression of endogenous K-Ras^G12D^ in MEFs decreased ROS production and increased Nrf2 as well as enzymes of the Nrf2 antioxidant program. The K-Ras^G12D^ mutation is commonly found in human pancreatic cancer [36]. Accordingly, higher NQO1 protein and lower levels of ROS biomarkers were detected in murine and human pancreatic intraepithelial neoplasia (PanIN) compared to normal tissue. The relevance of oncogene-mediated Nrf2 activation was proven in PanIN from Nrf2-deficient mice in which K-Ras^G12D^-induced proliferation and tumorigenesis was much less than in PanIN from wild-type (WT) mice.

A relatively novel system regulated by Nrf2 is the proteasome, a protease complex responsible for the degradation of proteins tagged with polyubiquitin chains [37]. The 26S proteasome consists of the catalytic 20S core subunit and the 19S regulatory particle, both consisting of different subunits. The 20S proteasome degrades oxidatively modified proteins and is activated upon mild oxidative stress [38]. The S5a subunit of the 19S proteasome and the *α*-5 subunit of the 20S proteasome were enhanced in colon tumors compared to the surrounding normal tissue [39]. The higher levels correlated with an elevated nuclear localization of Nrf2. Activation of Nrf2 by electrophilic stress in human colon cancer cell lines further elevated these subunits and increased TRAIL-mediated NF*κ*B activation leading to a protection against apoptosis [39].

Also upregulation of enzymes metabolizing xenobiotics will not always improve detoxification but increase the toxicity of xenobiotics as reviewed by Hayes et al. [40]. In fact, activated Nrf2 and upregulation of GSTP1 in hepatocarcinogenesis were the first hint to a supportive role of Nrf2 in cancer cells [41]. Thus, enhancement of Nrf2 and the resulting upregulation of multidrug resistance-associated proteins can help cancer cells to escape from chemotherapy [42, 43]. Chemoresistance has indeed been observed after treatment of breast cancer cells with tamoxifen [44] and of ovarian cancer cells with cisplatin [45] or other drugs [46]. Accordingly, knockdown of Nrf2 prevented resistance to tamoxifen in breast cancer cells [44] and resistance to doxorubicin in MEFs from Nrf2 knock-out (KO) mice [47].

However, Nrf2 is not activated in all types of cancer cells. It is even decreased in a high number of breast cancer cells compared to normal mammary epithelial cell lines. This coincides with variable but detectable levels of Keap1 and consistently increased mRNA and protein levels of Cul3, the ubiquitin ligase tagging Nrf2 for proteasomal degradation. Accordingly, downregulation of Cul3 in MCF-7 cells rescued Nrf2 and its targets [48]. A decrease of Nrf2 in the human breast cancer cells lines MDA-MB-231 and Hs578T is caused by silencing of the Keap1 RNA-destabilizing miR200a (miRNA) leading to a higher degradation of Nrf2 [49].

Taken together, cancer cells use all facets of the adaptive response to escape elimination. In consequence, under certain circumstances, the protective functions of Nrf2 can switch to procarcinogenic ones [7, 17, 28]. Nrf2 appears to be more active in some cancer cells and less in others, depending on the cell context, the nature of stress, and the cancer stage. Also Nrf2 targets may have dual roles in cancer which will here be discussed for the selenoproteins thioredoxin reductase-1 (TrxR1) [50, 51] and glutathione peroxidase-2 (GPx2) [52, 53].

## 3. TrxR1

Thioredoxin reductases (TrxRs) are a family of NADPH-dependent selenoflavoproteins (TrxR1, TrxR2, and TGR) present in almost all living cells (for reviews see [54, 55]). Together with thioredoxin (Trx) and NADPH, they build up the thioredoxin system. The system maintains a reducing environment in the cytosol, among others required for the redox regulation of gene expression via transcription factor activity. It, thus, is involved in DNA repair, angiogenesis, and inhibition of apoptosis. Moreover, during DNA synthesis Trx directly transduces electrons to ribonucleotide reductase which requires the continuous reduction of thereby oxidized Trx by TrxR. These functions underscore the pivotal role of TrxR in cell proliferation and survival [55, 56]. Due to its antioxidant function and its upregulation in cancer cell lines [57] and human gastrointestinal cancer tissue [58], TrxR was first expected to counteract malignant transformation. This hypothesis was supported by the fact that TrxR1 regulates the correct maturation of the tumor suppressor p53 [59, 60]. Furthermore, TrxR1 was the first selenoprotein identified as target of Nrf2 [50, 61] which at first glance was interpreted as support for a protective role [50, 62]. This might indeed hold true for the prevention of initiation of carcinogenesis in healthy cells.

However, it soon turned out that the beneficial effects of the Trx/TrxR system change to its opposite during the growth and progression phase of tumors. In fact, upregulation in cancer cells might also reflect the need of the enzyme for essential functions in cancer cells, the TrxR-dependent synthesis of deoxyribonucleotides [63]. Downregulation of TrxR1 by antisense RNA did not increase but inhibited growth of human hepatocarcinoma cells [64]. Also a knockdown of TrxR1 in lung carcinoma cells reversed their tumorigenicity and invasive potential in a xenograft model [65]. As underlying mechanism, the decreased expression of DNA polymerase *α* was supposed [66]. Alternatively, the antiapoptotic function of TrxR1 may come into play. Reduced Trx is required to inhibit apoptosis signal-regulating kinase (ASK) [67]. Lack of TrxR1 will prevent inhibition of ASK making the elimination of malignant cells by apoptosis possible. Not surprisingly, TrxRs have been suggested as potential targets for anticancer drugs [68, 69]. The inhibitory mechanism of such drugs often is the same as used for the dissociation of Nrf2 from Keap1, namely, thiol modification. Modification of the selenol in the active centre of TrxR1 indeed leads to an inhibition of the enzyme activity. On the other hand, the same drugs activate Nrf2 as evident from the upregulation of Nrf2 targets such as glutathione reductase, a glutathione peroxidase, and GST [69]. The thereby also upregulated TrxR1 might interfere with the inhibition of enzyme activity and facilitate cancer cell growth. This should be considered when selecting drugs to inhibit TrxR activity.

However, TrxR1 is not the only enzyme required for proliferation. Cells from mice with a differentiated hepatocyte-specific KO of TrxR1 were able to proliferate [70]. Proliferation of hepatocytes lacking TrxR1 was observed by *in vivo* staining [71] and MEFs from conditioned TrxR1 KO mouse embryos did not show impaired proliferation [72]. Thus, TrxR1 deletion appears to be compensated by another system. This most probably is the glutathione (GSH) system as demonstrated by a severely reduced replicative index if also GSH is depleted [71], and by an upregulation of GSH metabolizing enzymes in MEFs from conditioned TrxR1 KO mice [72]. The latter obviously is achieved in TrxR1-depleted cells by an activation of Nrf2 [73]. Deletion of TrxR1 in all parenchymal hepatocytes of mice resulted in a compensatory upregulation of Nrf2 targets including GSTs, GPx2, and sulfiredoxin [73]. It was, thus, concluded that ablation of *txnrd1* encoding TrxR1 would mimic oxidative challenge and switch on a constitutively active Nrf2 pathway [73]. What is activating Nrf2 when TrxR1 is absent is unclear, but it fits with the observations made with TrxR1 inhibitors (see above) and the upregulation of Nrf2 targets in moderate selenium deficiency in mice [74, 75]. A challenging hypothesis would be that the thioredoxin system maintains critical thiol groups in Keap1 in the reduced state and, thereby, prevents Nrf2 release. That Trx is able to reduce Cys151 in Keap1 has recently indeed been shown [76]. This way TrxR1 would serve as turn-off signal for the Nrf2 system.

In sum, a critical balance between Nrf2 and TrxR1 activities might exist which appears worth to be further investigated.

## 4. GPx2

The gastrointestinal glutathione peroxidase (GPx2) was first detected in the gastrointestinal system [77]. There it obviously plays a role in proliferating cells since its concentration is highest at crypt bases where proliferation takes place [78]. During human colon carcinogenesis, GPx2 is transiently increased with the highest expression in early adenoma and decreasing amounts in late stages of malignancy [78, 79]. It is also upregulated during the neoplastic transformation of squamous epithelial cells [80] and in lung adenocarcinomas of smokers [81] indicating that its expression is not restricted to the gastrointestinal system, but rather characteristic for rapidly dividing epithelial cells in general [82]. Evidences for a protective role are provided by genetically modified animals. A GPx2 KO rendered mice more susceptible to skin cancer development upon *γ*-irradiation [83]. Mice in which both GPx1 and GPx2 had been knocked out developed ileocolitis [84] and later intestinal cancer [85]. The lack of GPx2 was more detrimental, since one intact allele of GPx2 (but not of GPx1) was sufficient to prevent intestinal inflammation [82]. Mechanistic studies with HT29 cells with a stable downregulation of GPx2 by siRNA revealed that GPx2 suppresses COX2 expression and PGE_2_ production [86]. Furthermore, the siGPx2 cells exhibited an enhanced invasive potential and migrated faster in a wound healing assay [87]. Both effects obviously required the upregulated COX2 activity since celecoxib, a specific COX2 inhibitor, rescued the effects to the level observed in control cells. A protective role of GPx2 can also be inferred from its induction by Nrf2 [52]. Thus, the majority of the findings described so far characterize GPx2 as an anti-inflammatory enzyme.

The function of GPx2 as an anticarcinogenic enzyme is less clear, and evidence for an additional procarcinogenic role is increasing. Apoptosis at colonic crypt bases is drastically increased in GPx2 KO mice [88]. Inhibition of apoptosis may reflect the physiological function of GPx2 in crypt bases where it appears to support cell proliferation in the self-renewal of the intestinal mucosa. Cancer cells, however, will profit from not being eliminated by apoptosis which might well be the reason why the siGPx2 cells were not able to grow anchorage-independently and developed into much smaller tumors than WT cells when injected into nude mice [87]. Indeed, GPx2 expression is higher in proliferating cancer stem cells compared to their differentiated progeny [89]. Transcriptional regulation further points into a procarcinogenic direction. GPx2 is induced by ΔNp63 [90], a transcription factor necessary for cell proliferation, and its overexpression inhibited oxidant-mediated apoptosis [90]. Activation of the GPx2 promoter by *β*-catenin [91], which is the key mediator in the Wnt pathway and constitutively active in most of intestinal cancers, can again be interpreted controversially, either as an attempt to counteract carcinogenesis or to sustain cancer cell growth.

Some tentative answers may be derived from a study using an inflammation triggered model of colon carcinogenesis, the azoxymethane (AOM)/dextransulfate sodium (DSS) mouse model. In this study AOM/DSS treatment was combined with feeding WT and GPx2 KO mice a moderately Se-deficient, Se-adequate, and Se-supranutritional diet [92]. All AOM/DSS-treated mice developed colitis which was generally more severe in GPx2 KO mice than in WT mice under all Se states and especially high in moderate Se-deficiency. Inflammation and accordingly tumor formation were decreased under the Se supranutritional diet. Tumor numbers per animal tended to be higher in GPx2 KO mice at all selenium diets and were decreased by supranutritional selenium. In contrast, tumor size was smaller in GPx2 KO mice at the moderate selenium deficiency and in the supranutritional status, which correlates with the smaller tumors in nude mice developing from HT29 cells in which GPx2 was knocked down (see above). In the same experiment the effect of sulforaphane (SFN), a well-known Nrf2 activator, was tested. Surprisingly, it enhanced colitis in Se-poor WT and GPx2 KO mice but decreased it in Se-adequate mice to an identical score in both genotypes. The same dependency on selenium was observed for the reduction in the number of tumors and apoptotic cells by SFN in both GPx2 KO and WT mice. This indicates that SFN needs a selenoprotein or a selenium-dependent process to act beneficially. However, this protein cannot possibly be GPx2 since SFN effects were the same in WT and GPx2 KO mice. The responsible selenoprotein might rather be GPx1 which is upregulated in the intestine of GPx2 KO mice [88], but can only be synthesized when selenium is available. Taken together, there is an interplay between selenium and Nrf2 activators, but this appears to be much more complex than a synergistic upregulation by Nrf2-mediated transcriptional activation and subsequent translation of a specific selenoprotein.

In short, GPx2 appears to be a protective enzyme with pronounced anti-inflammatory potential and antiapoptotic capacity. Consequences of an upregulated GPx2 might, however, differ between healthy and malignant tissue. In healthy tissue, GPx2 is required to maintain the normal self-renewing of the gastrointestinal epithelium and, as part of the adaptive response, to depress inflammatory processes. This way GPx2 can inhibit initiation of carcinogenesis. But once a cell has been programmed to proliferate in an uncontrolled way, GPx2, *inter alia* by inhibiting apoptosis, supports further growth, which does not appear particularly beneficial. This view is in line with the clinical study described in the beginning which revealed a decrease of esophageal cancer incidence by selenium only in younger but not in older participants [5]. Similarly, in recent N-nitrosomethylbenzylamine-induced esophageal squamous cell carcinoma (ESCC) study, the numbers of dysplasia and ESCC were significantly lower in rats on supplementation with selenium and vitamin E only during the early stage of tumor development or during the entire experimental period but not during the late stage [93].

## 5. Conclusions

The benefit of upregulation/activation of the Nrf2 pathway of the selenoproteins TrxR1 and GPx2 differs in healthy and in cancer cells. Via its physiological role in a program maintaining the cellular redox state and *inter alia* the endogenous defense systems and by preventing apoptosis and damage by a dysregulated redox homeostasis, Nrf2 might contribute to the prevention of cancer initiation in healthy cells.

The vital function of TrxR1 is explained by its role in the replication and proliferation of developing healthy cells. The physiological function of GPx2 appears to support proliferation of crypt base epithelial cells in the self-renewal of the gastrointestinal epithelium. Its antiapoptotic and anti-inflammatory properties might help to inhibit the initiation and promotion of carcinogenesis by proinflammatory mediators.

However, if a cell has been transformed into a malignant cell and the carcinogenic process has started, the cancer cells will equally profit from the protective roles of TrxR1, GPx2, and other Nrf2 programs and, accordingly, will grow unhampered.
